# Editorial: CRISPR and beyond: Cutting-edge technologies for gene correction in therapeutic applications

**DOI:** 10.3389/fgeed.2023.1203864

**Published:** 2023-04-20

**Authors:** Ayal Hendel, Rasmus O. Bak

**Affiliations:** ^1^ The Institute for Advanced Materials and Nanotechnology, The Mina and Everard Goodman Faculty of Life Sciences, Bar-Ilan University, Ramat Gan, Israel; ^2^ Department of Biomedicine, Aarhus University, Aarhus, Denmark

**Keywords:** gene editing, gene correction, CRISPR/Cas, HDR, nucleases, prime editing, base editing, perspective

Gene editing promises the ultimate cure for genetic diseases by directly correcting disease-causing variants. However, the first clinical trials have chased the “low hanging fruit” using editing strategies that rely on gene disruption by introducing double-strand DNA breaks that lead to insertions and deletions (indels) by the NHEJ pathway. Since NHEJ is constitutively active throughout the cell cycle and the default DNA repair pathway, this is by far the most efficient type of conventional gene editing as opposed to homology-directed repair (HDR). HDR relies on delivery of an exogenous repair template and this pathway is active only in the S and G2 phases of the cell cycle. These two parameters constitute challenges in clinical use of HDR since exogenous DNA is toxic in most therapeutically relevant cell types and since the inherent competition between NHEJ and HDR can be a bottleneck. However, HDR benefits from enabling precise edits to be made to the genome, thereby representing true gene editing with control over the outcome. Still, in both these modalities the DNA breaks are considered a potential source of genotoxicity due to the possibility of off-target edits and chromosomal aberrations such as translocations and chromothripsis. Next-generation gene editing tools like Base and Prime Editing that rely on DNA single strand nicking reduce the risk of such harmful events but are still limited in the scope of the edits they can generate (Anzalone et al., 2020). The newest types of editors based on CRISPR-associated transposases or CRISPR-directed integrases facilitate larger edits but are still under development and immature for clinical implementation (Yarnall et al., 2022; Tou et al., 2023). This rapidly developing toolbox is expected to broaden the application of CRISPR-based tools and other site-specific engineered nucleases to cure human disease. However, on this venture of realizing precise gene correction there are several unanswered questions and challenges to overcome, some of which we hope to address with this Research Topic on Therapeutic Gene Correction Strategies Based on CRISPR Systems or Other Engineered Site-specific Nucleases. This Research Topic covers a selection of contributions including significant scientific advances in precise genetic engineering as well as expert perspectives on recent advances.

The article by Lu et al. summarizes the recent improvements in nuclease and nickase genome editing approaches for the treatment of genetic diseases (Lu et al.). Furthermore, it highlights the major challenges in the translation of these approaches into clinical applications. The article by Reshetnikov et al. reviews the status of Base Editors for the correction of point mutations in monogenic diseases (Reshetnikov et al.). Cytosine and adenine Base Editors convert C•G to T•A and A•T to G•C, respectively and these tools are described in detail along thorough overviews on studies using these base editors *in vivo* and *in vitro*/*ex vivo* for monogenic retinal, neuromuscular, blood, neurodegenerative, and metabolic disorders.

Transcription activator-like effector domain (TALE) base editors (BEs) are a recent exciting addition to the genome editing toolbox. Boyne et al. in their original paper define the optimal TALE-BEs activity window and demonstrate the feasibility of efficient multiplex gene engineering using a combination of two different molecular tools, a nuclease and a base editor (Boyne et al.). Using such multiplex strategy has numerous important benefits, including better control over the editing outcomes by avoiding the occurrence of translocations that commonly arise when multiple nucleases are employed simultaneously, and it offers the opportunity to achieve more than just multiple knock-outs, as gene knock-ins can also be accomplished at the nuclease target site.

In extension of this, Wolff et al. introduces a new tool for high-efficiency Prime Editing termed piggyPrime (Wolff et al.). This tool makes use of the piggyBac transposon system to facilitate genomic integration of all the Prime Editing genetic components in cells to allow accumulation of prime edits over time leading to up to 100% of targeted alleles in some cell lines. This enables effective generation of transgenic cell lines to model disease-causing genetic variants.

Usher et al. similarly explores the generation of model cell lines carrying disease-causing variants but make use of conventional CRISPR/Cas HDR-based gene editing to install variants into the genome of cell lines (Usher et al.). By compiling data from 95 transfections, they compare HDR parameters such as donor template modifications, concentration, HDR enhancers, and cold shock. They also find that guideRNA efficiency prediction by online algorithms correlate poorly with activity in cells, and they present a workflow for designing and performing gene editing experiments to generate and characterize disease model clonal lines.

The articles by Houghton et al. and Ravendran et al. both describe gene editing approaches to fight monogenic inborn errors of immunity (IEI). Ravendran et al. reviews the DOCK8 immunodeficiency syndrome, which is a type of autosomal recessive hyper IgE syndrome caused by defects in the DOCK8 gene. The authors outline different genome editing strategies that might be applied to cure this devastating immunodeficiency syndrome (Ravendran et al.). Houghton et al. focus on another IEI, X linked lymphoproliferative disease (XLP), caused by mutations or deletions in the SH2D1A gene. The study compares the use of TALENs, CRISPR/Cas9, and CRISPR/Cas12a in combination with AAV6 repair template delivery (Houghton et al.). The components target exon 1 close to the start codon to facilitate integration of a near-full SH2D1A cDNA to be physiologically expressed and regulated by the endogenous promoter. The study shows integration frequencies around 30%-50% in T cells and that this restores SH2D1A gene expression and immune function in patient T cells to levels observed in healthy controls.

While genome editing is a very promising technology, it could, in theory, cause safety issues. Wienert and Cromer discuss the potential for unintended effects of CRISPR nuclease activity in human clinical trials (Wienert and Cromer). In their review they summarize the current sequencing-based solutions that may be able to detect these small and large-scale unintended genome editing effects even at low frequencies of occurrence. They highlight the safety and ethical concerns surrounding *in vivo* delivery of CRISPR tools and the potential for unintended editing in unintended cell types, which could enable germline transmission. Finally, they also outline some advanced potential mitigation strategies that will ensure that the safety of CRISPR keeps pace with its efficacy.

Schmidt et al. and Atkins et al. similarly cover additional important aspects of unintended on- and off-target editing outcomes. Schmidt et al. evaluate on- and off-target editing outcomes in CCR5 CRISPR-Cas9-targeted Mauritian cynomolgus macaque embryos using whole genome sequencing (WGS) analysis (Schmidt et al.). In this first report of WGS analysis of CRISPR-Cas9-targeted non-human primate embryonic cells they identify large deletions at the on-target site and *de novo* mutations at predicted CRISPR/Cas9 off-target sites. These data clearly demonstrate that comprehensive sequencing-based methods are warranted for evaluating editing outcomes in primate embryos and therefore also highlights the risks in human embryo editing. Atkins et al. examine in their review the strengths and limitations of the different classes of off-target cleavage detection techniques (Atkins et al.). Furthermore, they also discuss the clinical relevance of these techniques in the context of assessing the safety of novel CRISPR/Cas9 HIV curative strategies that are currently examined in clinical trials.

In summary, we hope that the original papers and reviews we had the privilege of including in our Research Topic will be a further step in bringing these therapeutic gene correction strategies to patients in need.
